# The Law Affecting Medical Practitioners.—IV

**Published:** 1902-12-27

**Authors:** J. E. R. Stephens

**Affiliations:** Barrister-at-Law, Author of "Digest of Public Health Cases"


### The Institutional Workshop.

### THE LAW AFFECTING MEDICAL PRACTITIONERS.—IV.

By J. E. R. Stephens, Barrister-at-Law, Author of "Digest of Public Health Cases."

### Copyright of Writings and Lectures of Medical Men.

The term copyright includes two rights—the right exists in every unpublished innocent production of art, literature, or science, and may be termed copyright before publication. It exists *a fortiori* in every such published production, and may be termed copyright after publication. In reference to the first of these, viz., copyright before publication, it may be said generally that when any material has embodied the ideas which spring up in the mind of the author or the artist, the law protects their privacy until the material has itself been, by its owner, given to the world. In the language of Mr. Justice Yates (4 Burr. 2378), "A man has certainly the right to consider whether he will make his ideas public, or commit them only to the sight of his friends; in that state a manuscript is in every sense his peculiar property, and no man can take it from him, or make a use of it which he has not authorised, without being guilty of a violation of his property. And as every author or proprietor of a manuscript has a right to determine whether he will publish it or not, he has a right to the first publication, and whoever deprives him of that privilege is guilty of a manifest wrong, and the Court have a right to stop it."

In the case of *Abernethy* v. *Hutchinson* (3 L. J. Ch. 209-219) Mr. Abernethy had filed a bill to restrain the publication in the *Lancet* of certain lectures delivered by him orally in St. Bartholomew's Hospital. Lord Eldon refused to grant the injunction, because the plaintiff was unable to swear that his whole lecture had been reduced to writing at the date of its delivery. Lord Eldon observed: "Where the lecture is orally delivered, it is difficult to say that an injunction can be granted upon the same principle upon which literary composition is protected, because the Court must be satisfied that the publication complained of is an invasion of the work, and this can only be done by comparing the composition with the piracy." His lordship, however, upon a second argument, granted the relief prayed, but on a different ground, viz., that of a breach of an implied contract between the lecturer and his audience, that the latter would do nothing more than listen to the lecture for their instruction.

On publication, no more passes to the public than an unlimited use of every advantage that the purchaser can reap from the doctrines and sentiments which the work contains. The property in the composition does not pass; for those things which are peculiarly and appropriately his must remain his until he agrees or consents to part with them by compact or donation, because no man can deprive him of them without his approbation.

Lectures are generally more or less literary productions—frequently the result of much thought and research. They are continually being published in the form of books and pamphlets—suth publications being in many cases of great value. If a lecture has been reduced wholly or partially into writing, the author has clearly a right of property in it; and the Court, when called upon to restrain the publication of such a lecture, will compare the original composition with the piracy. The admission of persons to hear such a lecture affords no presumption that the speaker intends to give them a right to publish the information they may acquire. When persons are admitted as pupils or otherwise to listen to lectures orally delivered, although they may go to the extent, if desirous and capable, of taking down the whole by means of shorthand, yet they can do that only for the purpose of their own information, and not for publication. A leading case on this point is that of *Nicols* v. *Pitman* (26 Ch. D. 374). Nicols, an author and a lecturer upon various scientific subjects, delivered from memory, though it was in manuscript, a lecturer at the Working Men's College upon "The Dog as the Friend of Man." The audience were admitted to the room by tickets issued gratuitously by the committee of the college. Mr. Pitman, the author of a system of shorthand writing and the publisher of works intended for instruction in the art of shorthand writing, attended the lecture and took notes, nearly *verbatim*, in shorthand of it, and afterwards published the lecture in his monthly periodical *The Phonographic Lecturer*. On a motion for an injunction to restrain the publication, it was held that where a lecture of this kind is delivered to an audience, limited and admitted by tickets, the understanding between the lecturer and the audience is that, whether the lecture has been committed to writing beforehand or not, the audience are quite at liberty to take the fullest notes for their own personal purposes, but they are not at liberty to use them afterwards for the purpose of publishing the lecture for profit; and the publication of the lecture in shorthand characters is not regarded as being different in any material sense from any other, and an injunction was granted accordingly. So where a professor of a university delivers orally in his class-room lectures which are his own literary composition, no person is entitled to republish them without the permission of the author. The copyright in lectures is specially protected by the 5 & 6 Will. IV., c. 65. This statute provides that the author of any lecture, or the person to whom he has sold or otherwise conveyed the copy in order to deliver the same in any school, seminary, institution, or other place, or for any other purpose, shall have the sole right and liberty of printing and publishing such lectures; and that if any person shall, by taking down the same in shorthand, or otherwise in writing, or in any other way, obtain or make a copy of such lecture, and shall print or lithograph or otherwise copy and publish the same, or cause the same to be printed, etc., without leave of the author thereof, or of the person to whom the author has sold or otherwise conveyed the same, and every person who, knowing the same to have been printed or copied and published without such consent, shall sell, publish, or expose to sale, or cause to be sold, published, or exposed to sale, any such lecture, shall forfeit such printed or otherwise copied lecture or parts thereof, together with one penny for every sheet thereof, which shall be found in his custody, the one moiety thereof to His Majesty, his heirs, or successors, and the other moiety thereof to any person who shall sue for the same. The Act further declares that no person allowed for a certain fee and reward, or otherwise to attend and be present at any lecture delivered at any place, shall be deemed and taken to be licensed, or to have leave to print, copy, and publish such lecture on account merely of having permission to attend the delivery. The following lectures are excluded from the provisions of the Act:—Lectures of the delivery of which notice in writing shall not have been given two days previously to two justices living within five miles of the place of delivery, and those delivered in any university, or public school, or college, or any public foundation, or by any individual in virtue of, or according to any gift, endowment, or foundation. In consequence of these provisions few lectures are protected by the Act, for seldom is the requisite notice given. The notice must be given every time such lecture is delivered, and therefore the omission in any one instance to give the requisite notice would render any person at liberty to obtain a copy, which the lecturer would be unable to prevent his publishing.

The important question with regard to any lecture as to which the statutory notice has not been given is, Has it been published? If it has been published in print, it will receive statutory protection as a book under 5 and 6 Vict. c. 45. If the lecture has not been printed the question is whether the circumstances under which it was orally delivered amount to a publication. A lecturer who addresses himself to the public generally, without distinction of persons or selection and restriction of hearers, abandons his ideas and words to the use of the public at large, that is, he publishes them. On the other hand, where it is matter either of express contract or implied condition that the audience are admitted for the purpose of receiving instruction or amusement, and not in order that they may take a full note of what they hear and publish it for their own profit and the information of the public at large, publication does not take place. Where a lecture is delivered on behalf of the university and as the authorised exposition of the university teaching it may be that there is publication. But the decision of the House of Lords in *Caird* v. *Sime* (12 A.C. 326) shows that the lectures of a university professor are not necessarily published by delivery to his class, indeed, are probably delivered under such circumstances that no republication can take place without his consent.

